# Triple-marker cardiac MRI detects sequential tissue changes of healing myocardium after a hydrogel-based therapy

**DOI:** 10.1038/s41598-019-55864-7

**Published:** 2019-12-18

**Authors:** Maaike van den Boomen, Hanne B. Kause, Hans C. van Assen, Patricia Y. W. Dankers, Carlijn V. C. Bouten, Katrien Vandoorne

**Affiliations:** 1 Department of Biomedical Engineering, Cell-Matrix Interaction for Cardiovascular Tissue Regeneration, Eindhoven University of Technology, Eindhoven, The Netherlands; 2 Department of Radiology, University Medical Center Groningen, University of Groningen, Groningen, Netherlands; 3 Department of Radiology, Athinoula A. Martinos Center for Biomedical Imaging, Massachusetts General Hospital, Harvard Medical School, Charlestown, MA United States; 4 Department of Electrical Engineering, Eindhoven University of Technology, Eindhoven, The Netherlands; 5 Institute for Complex Molecular Systems (ICMS), Eindhoven University of Technology, Eindhoven, The Netherlands; 6 Department of Biomedical Engineering, Laboratory of Chemical Biology, Eindhoven University of Technology, Eindhoven, The Netherlands

**Keywords:** Molecular imaging, Cardiac regeneration

## Abstract

Regenerative therapies based on injectable biomaterials, hold an unparalleled potential for treating myocardial ischemia. Yet, noninvasive evaluation of their efficacy has been lagging behind. Here, we report the development and longitudinal application of multiparametric cardiac magnetic resonance imaging (MRI) to evaluate a hydrogel-based cardiac regenerative therapy. A pH-switchable hydrogel was loaded with slow releasing insulin growth factor 1 and vascular endothelial growth factor, followed by intramyocardial injection in a mouse model of ischemia reperfusion injury. Longitudinal cardiac MRI assessed three hallmarks of cardiac regeneration: angiogenesis, resolution of fibrosis and (re)muscularization after infarction. The multiparametric approach contained dynamic contrast enhanced MRI that measured improved vessel features by assessing fractional blood volume and permeability*surface area product, T_1_-mapping that displayed reduced fibrosis, and tagging MRI that showed improved regional myocardial strain in hydrogel treated infarcts. Finally, standard volumetric MRI demonstrated improved left ventricular functioning in hydrogel treated mice followed over time. Histology confirmed MR-based vessel features and fibrotic measurements. Our novel triple-marker strategy enabled detection of ameliorated regeneration in hydrogel treated hearts highlighting the translational potential of these longitudinal MRI approaches.

## Introduction

Cardiovascular diseases, including myocardial infarction (MI), remain the leading cause of mortality across the world^1^. MI is caused by an occlusion of a coronary artery, resulting in cardiomyocyte cell death due to insufficient nutrient and oxygen supply. This primary injury provokes a cascade of events that eventually could lead to cardiac remodeling and heart failure. Firstly, local cell death induces the inflammatory phase of infarct healing, starting shortly after the MI. Blood vessels become permeable allowing inflammatory cells to clean the wound of dead cells and debris^2^. Secondly, the proliferative phase begins several days after MI. Activated myofibroblasts produce extracellular matrix proteins accompanied by angiogenesis at the infarct borders. Finally, during maturation phase a collagen-rich scar is formed, replacing viable myocardium and stabilizing the ventricular wall. Because of the loss of healthy cardiomyocytes by scar formation, the non-ischemic remote myocardium undergoes hypertrophy to compensate for the reduced contractile force development after acute MI. Chronically, adverse cardiac remodeling deteriorates cardiac function resulting in heart failure development^3^.

Several regenerative approaches attempting to improve outcome or even regenerate the damaged heart after MI, are still in their infancy. Highly promising strategies to regenerate viable myocardium by grafting or activating stem and/or progenitor cells or by re-entering cardiac myocytes into the cell cycle are still an unfulfilled ambition^4^. Transplantation of adult stem cells improve short-term cardiac function post-MI, yet excreted paracrine factors, such as vascular endothelial growth factor (VEGF) and insulin growth factor 1 (IGF1) account for this therapeutic improvement^4^. VEGF is a cytokine involved in the formation of new blood vessels or angiogenesis^5^. IGF1 is a key regulator of cell proliferation and survival, differentiation, and metabolism with cardioprotective properties^6–8^.

A promising regenerative strategy involves the application of biomaterials as scaffolds that can stimulate repair and regeneration^9,10^. Injectable hydrogels have been proposed and tested to deliver biological agents post-MI^11,12^. The use of hydrogels could create a three-dimensional (3D) matrix, resembling the endogenous extracellular matrix^11^ and sustained delivery of entrapped growth factors^13^. Here, we used a hydrogel based on supramolecular ureido-pyrimidinone (UPy) moieties coupled to poly(ethylene glycol) chains (UPy-hydrogel) loaded with VEGF and IGF1 (UPy^GF^-hydrogel). Previously, we showed that a local catheter injection of this UPy-hydrogel into porcine hearts after MI allowed local release of hepatocyte growth factor (HGF) and IGF1 reducing the size of the infarct scar^14^. The transient network of the UPy-hydrogel was shown to be pH-responsive, which enables injection in the solution state at basic pH followed by formation of a transient network at neutral tissue pH. Rheology and viscosity measurements of the UPy-hydrogel have been previously described^14^. Additionally, we recently demonstrated the successful *in vivo* identification of an injected UPy-hydrogel based on a UPy-unit modified with gadolinium (Gd) complex by magnetic resonance imaging (MRI)^15^. In the present study an UPy^GF^-hydrogel was locally injected to deliver VEGF and IGF1 in order to promote tissue repair to improve outcome after MI^5,7^.

Cardiac MRI is a powerful translational technology^16^ that could improve the assessment of cardiac healing aspects of novel regenerative treatments. Each novel cardiac regenerative therapy changes the complex spatial and temporal tissue dynamics of infarct healing after MI. Yet, *in vivo* cardiac imaging methods to serially evaluate these tissue changes with respect to the efficacy of novel regenerative treatments, are still an unmet need^17^. Cardiac MRI has previously reported on each of these hallmarks of interest separately^18^. First, albumin-based dynamic contrast enhanced (DCE) MRI allows noninvasive assessment of vascular density and permeability, which are both vessel features that could indicate angiogenesis^19,20^. Second, mapping T1 relaxation times supports *in vivo* evaluation of fibrosis^21,22^. Lastly, tagging MRI to measure strain, indicates local (re)muscularization^23^.

The novelty of this study is the combinatorial assessment of three hallmarks of regeneration in one single MRI session, namely angiogenesis, resolution of fibrosis and remuscularization simultaneously^18^. This integrated approach offers tremendous benefits by assessing dynamic cardiac regeneration and remodeling processes at high spatial and temporal resolution. We envision that such multiparametric platforms will enable simultaneous noninvasive studies of novel regenerative cardiac therapies by evaluating cardiac hallmarks in the clinics, which gives improved insights about the efficacy those therapies. To evaluate this triple-marker approach, we noninvasively assessed a novel local cardiac treatment of a UPy^GF^-hydrogel in a murine ischemia reperfusion (I/R) injury model, which is expected to alter each of these biomarkers based on each of their independent regenerative capacities.

## Results

### UPy^GF^-hydrogel specifications and tracking

**Figure 1:**
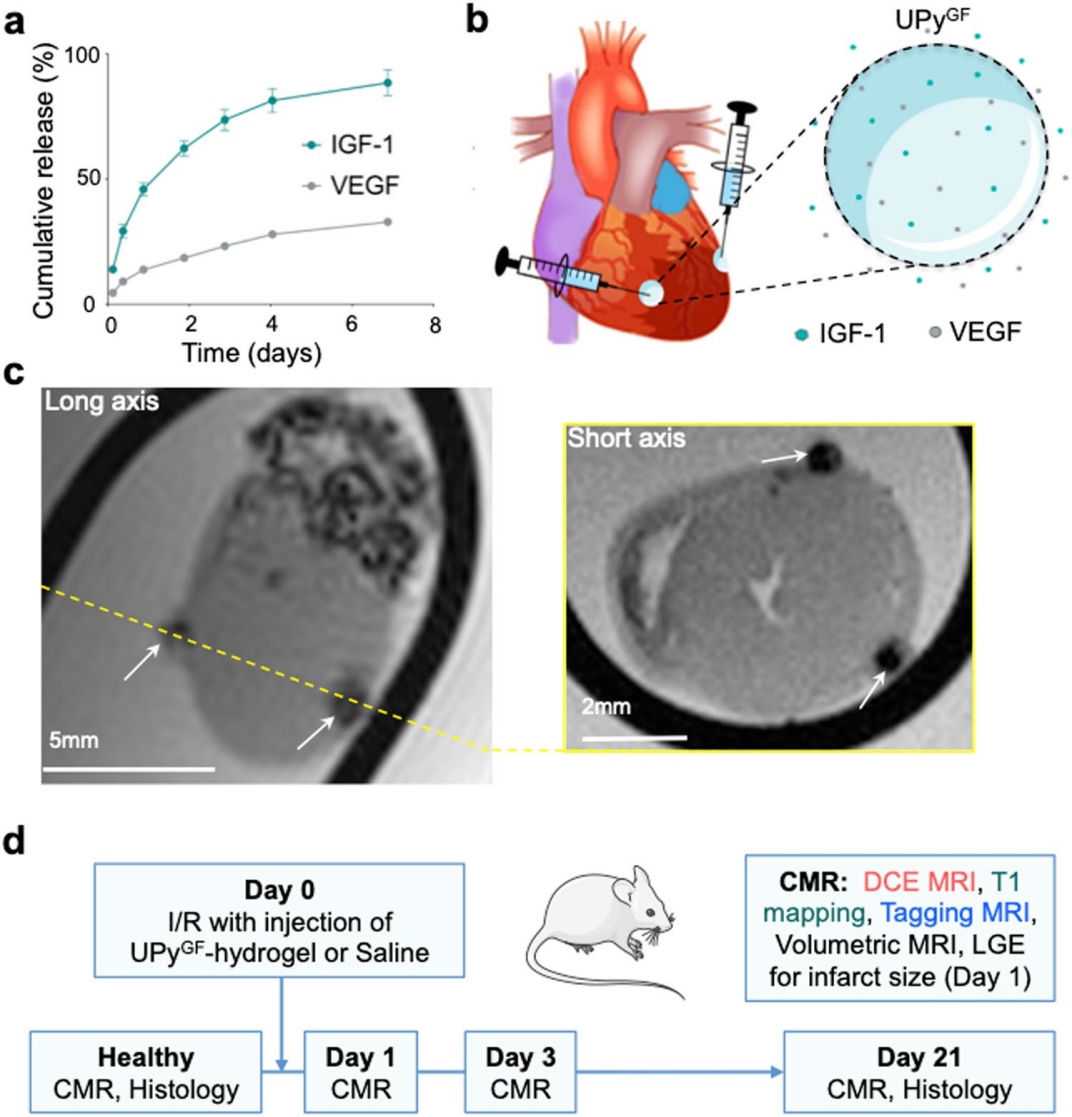
UPy^GF^-hydrogel characterization and *in vivo* setup. (**a)** *In vitro* release of IGF1/VEGF embedded in the UPy^GF^-hydrogel by daily collection of medium at 37 °C over one week (mean ± s.e.m, n = 4 measurements). (**b**) Localization of intramyocardial delivery of UPy^GF^-hydrogel 2 min before reperfusion in a mouse model of myocardial ischemia reperfusion (I/R). pH-switchable UPy^GF^-hydrogel loaded with insulin-like growth factor 1 (IGF1) and vascular endothelial growth factor (VEGF) in a solution state during basic conditions. After local injection, the UPy^GF^-hydrogel slowly released insulin-like growth factor 1 (IGF1) and vascular endothelial growth factor (VEGF). (**c**) Proof-of-concept *ex vivo* T_2_* MRI to verify intramyocardial UPy^GF^-hydrogel injections (arrows) labeled with ultrasmall superparamagnetic iron oxide at an infarcted heart 30 min after I/R. (**d**) *In vivo* experimental set-up of serial cardiac magnetic resonance imaging (MRI) and histology of hearts before and after I/R. The illustration of the heart (1b) and the mouse (1d) were modified from Servier Medical Art (http://smart.servier.com/), licensed under a Creative Common Attribution 3.0 Generic License.

*In vitro* release of IGF1 was sustained from the UPy^GF^-hydrogel for the first 7 days until 84 ± 5%, and the release of VEGF approached 33 ± 1% after 7 days (Fig. 1a). This indicated that incorporation of these growth factors in the UPy^GF^-hydrogel enabled a sustained release for the active phases of cardiac repair^3^. The UPy^GF^-hydrogel loaded with IGF1 and VEGF was intramyocardially injected at two locations at the border of the ischemic area 2 min before reperfusion (Fig. 1b). In order to verify its intramyocardial localization, the UPy^GF^-hydrogel was loaded with ultrasmall superparamagnetic iron oxide (USPIO). During I/R injury, USPIO-loaded UPy^GF^-hydrogel was locally injected at the border zone. *Ex vivo* T_2_*-weighted MR imaging of isolated hearts successfully identified the two injection sites (Fig. 1c).

### Implementation of *in vivo* triple-marker cardiac MRI in healthy hearts

**Figure 2:**
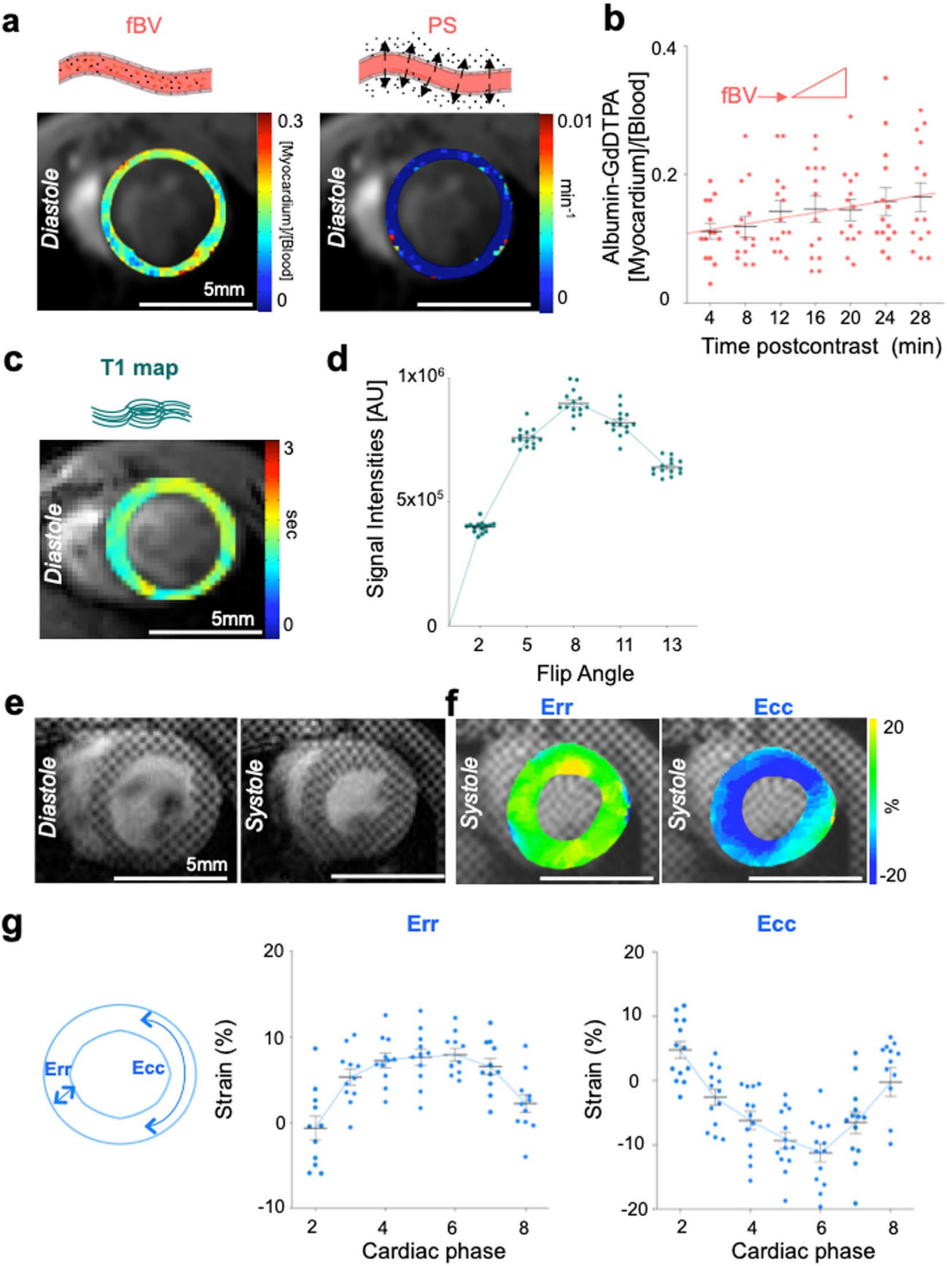
Noninvasive multiparametric cardiac MRI of healthy murine hearts. (**a**) Postcontrast parametric maps of fractional blood volume (fBV) and permeability*surface area product (PS) calculated from albumin-based dynamic contrast-enhanced MRI in hearts of healthy mice. (**b**) Contrast agent accumulation in the consecutive MR images for fBV (intercept at time 0) and PS (=slope) in control (n = 14). (**c**) Pixel-based parametric T_1_ map of a healthy heart. (**d**) Flip angle based signal intensities to calculate T_1_ cardiac maps (n = 14). (**e**) Tagging MR images of the healthy heart during diastole and systole. (**f**) Parametric maps of circumferential strain (Ecc) and radial strain (Err). (**g**) Individual values of circumferential and radial strain for 8 different cardiac phases (n = 13). Each dot represents an individual mouse; bars represent mean ± s.e.m.

The hallmarks of cardiac repair were assessed *in vivo* before and at several timepoints after I/R injury by MRI as shown in the study outline (Fig. 1d). Firstly, to quantify vascular repair processes, intravenous injection of paramagnetically-labeled albumin was followed by DCE MRI (Fig. 2a,b). Cardiac MRI-derived parametric maps confirmed healthy myocardial fractional blood volume (fBV) of 0.10 ± 0.01, a measure for microvascular density and permeability*surface area product (PS) of 0.0014 ± 0.0004 min^−1^, detecting endothelial barrier function (Fig. 2a)^19,20^. The fBV was calculated from the initial enhancement by the macromolecular albumin immediately after injection (intercept with the y-axis), when albumin was confined to the blood vessels. The PS was derived from the rate of change in contrast concentration in the myocardium with time (Fig. 2b). Secondly, the degree of myocardial fibrosis was measured by mapping T_1_ relaxation times from pre-contrast signal intensities at variable flip angles of cardiac MRI. Mean T_1_ value of healthy myocardium measured 1.541 ± 0.030 s (Fig. 2c,d), which is similar to previously published data^19,24^. Thirdly, tagging MRI was performed with local spatial tag frequency estimation to analyze regional myocardial function^23,25^ to determine changes in the peak myocardial strain (Fig. 2e; Supplementary Movie 1). Healthy peak strains in the myocardium were for 9.1 ± 0.8% for radial strain (Err) and −12.8 ± 1.4% for circumferential strain (Ecc) (Fig. 2f,g).

### DCE MRI reveals improved angiogenesis in hydrogel treated infarcts

**Figure 3:**
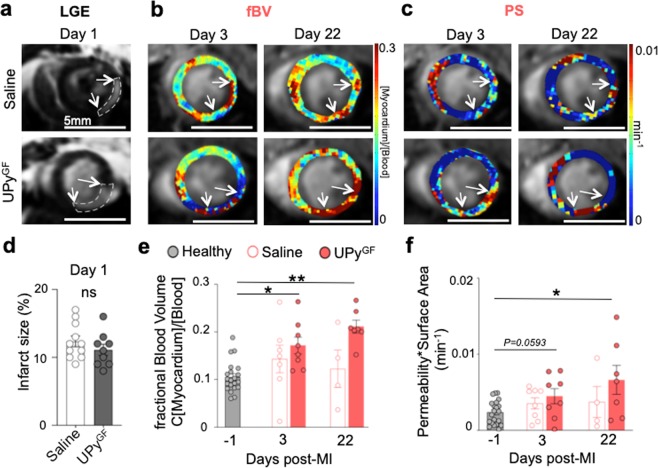
Serial contrast-enhanced cardiac MRI shows altered vascular features during cardiac repair in UPy^GF^-hydrogel injected infarcts. (**a**) Late gadolinium enhanced (LGE) MRI with arrows showing the infarct day 1 post-MI. (**b**) Postcontrast parametric maps of fractional blood volume (fBV) and (**c**) permeability*surface area product (PS) calculated from albumin-based dynamic contrast-enhanced MRI. (**d**) Infarct size one day after I/R. (**e**) Mean fBV and, (**f**) mean PS in healthy myocardium and in saline and UPy^GF^-hydrogel injected infarcts day 3 and day 22 post-infarction. Each dot represents an individual mouse; healthy mice (n = 20), saline injected mice (n = 11, day 1; n = 7, day 3; n = 4, day 22), UPy^GF^ injected mice (n = 9. day 1; n = 8, day 3; n = 7, day 22); bars represent mean ± s.e.m; Mann-Whitney U test; *P < 0.05, **P < 0.01.

At day 1 after I/R, GdDTPA was intravenously injected to verify infarct size by late gadolinium enhanced (LGE) MRI (Fig. 3a). Serially at day 3 and 22 after I/R, macromolecular GdDTPA-albumin-RhoB was administered to characterize vascular parameters fBV (Fig. 3b) and PS (Fig. 3c). LGE MRI showed a small infarct size for saline (12.4 ± 1.2%) and UPy^GF^-hydrogel (11.1 ± 1.1%) treated mice (Fig. 3d) similar to previously published data^26,27^. This I/R injury model is clinically very relevant^27,28^ and the similar infarct size allowed further comparison of consequent cardiac repair.

To follow the effect of slow releasing IGF1 and VEGF from the UPy^GF^-hydrogel at the border zone of the infarcted myocardium, vascular features were followed by albumin-based DCE MRI. Longitudinal follow-up showed an increase of fBV at day 3 (0.17 ± 0.02) and at day 22 (0.21 ± 0.01) in the reperfused myocardium treated with the UPy^GF^-hydrogel compared to healthy myocardium (0.11 ± 0.01). The increase of fBV was not significantly different at day 3 (0.14 ± 0.03) and at day 22 (0.12 ± 0.04) after I/R in the saline treated reperfused myocardium compared to healthy myocardium (Fig. 3e). Furthermore, the endothelial barrier function was analyzed. The PS values in the infarcted area at day 3 in saline-treated (4.2 ± 0.9 × 10^–3^min^−1^) and UPy^GF^-hydrogel treated (3.6 ± 1.1 × 10^–3^min^−1^; *P* *=* *0.0593*) appeared higher than healthy PS values, but were not significantly different from healthy myocardium. Additionally at day 22, PS values of saline-treated reperfused myocardium (3.6 ± 1.4 × 10^–3^min^−1^) remained similar to the healthy myocardium. Only at day 22 after I/R a significant increase of PS was demonstrated in the UPy^GF^-hydrogel treated (6.6 ± 1.7 × 10^–3^min^−1^) compared to healthy myocardium (Fig. 3f). This indicated improved vascular density and enhanced angiogenesis at the UPy^GF^-hydrogel treated infarcts.

### T_1_-mapping demonstrates mitigated cardiac fibrosis in hydrogel treated infarcts

**Figure 4:**
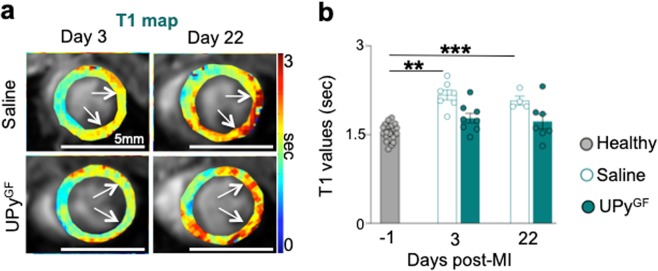
Longitudinal T_1_ mapping uncovers reduced fibrosis throughout cardiac healing in UPy^GF^-hydrogel injected infarcts. (**a**) Longitudinal T_1_ mapping of healthy hearts and hearts after I/R injected with saline or UPy^GF^-hydrogel. (**b**) T_1_ values. Each dot represents an individual mouse; n = 20 for healthy, healthy mice (n = 20), saline injected mice (n = 7, day 3; n = 4 day 22), UPy^GF^ injected mice (n = 8, day 3; n = 7, day 22); bars represent mean ± s.e.m.; Mann-Whitney U test; **P < 0.01; *** P < 0.001.

Fibrosis is inherent to scar tissue formation and the fibrotic response of the tissue may be altered by UPy^GF^-hydrogel injection after I/R. At day 3 post-infarction, T_1_ values of both saline (2.172 ± 0.008 s) and UPy^GF^-hydrogel (1.780 ± 0.083 s) injected ischemic myocardium were increased compared to T_1_ values of healthy myocardium (1.541 ± 0.030 s) but only the saline group was significantly different (Fig. 4a,b). At day 22 after I/R, the saline-injected infarcts still demonstrated a significantly increased T_1_ (2.077 ± 0.075 s), indicating increased fibrosis. At day 22 after I/R, T_1_ values of UPy^GF^-hydrogel treated infarcts (1.724 ± 0.123 s) did not increase further and remained non-significantly different from healthy myocardium (Fig. 4a,b). This suggests reduced fibrosis in infarct treated with UPy^GF^-hydrogel.

### Tagging MRI shows enhanced regional myocardial strain in hydrogel treated hearts

**Figure 5:**
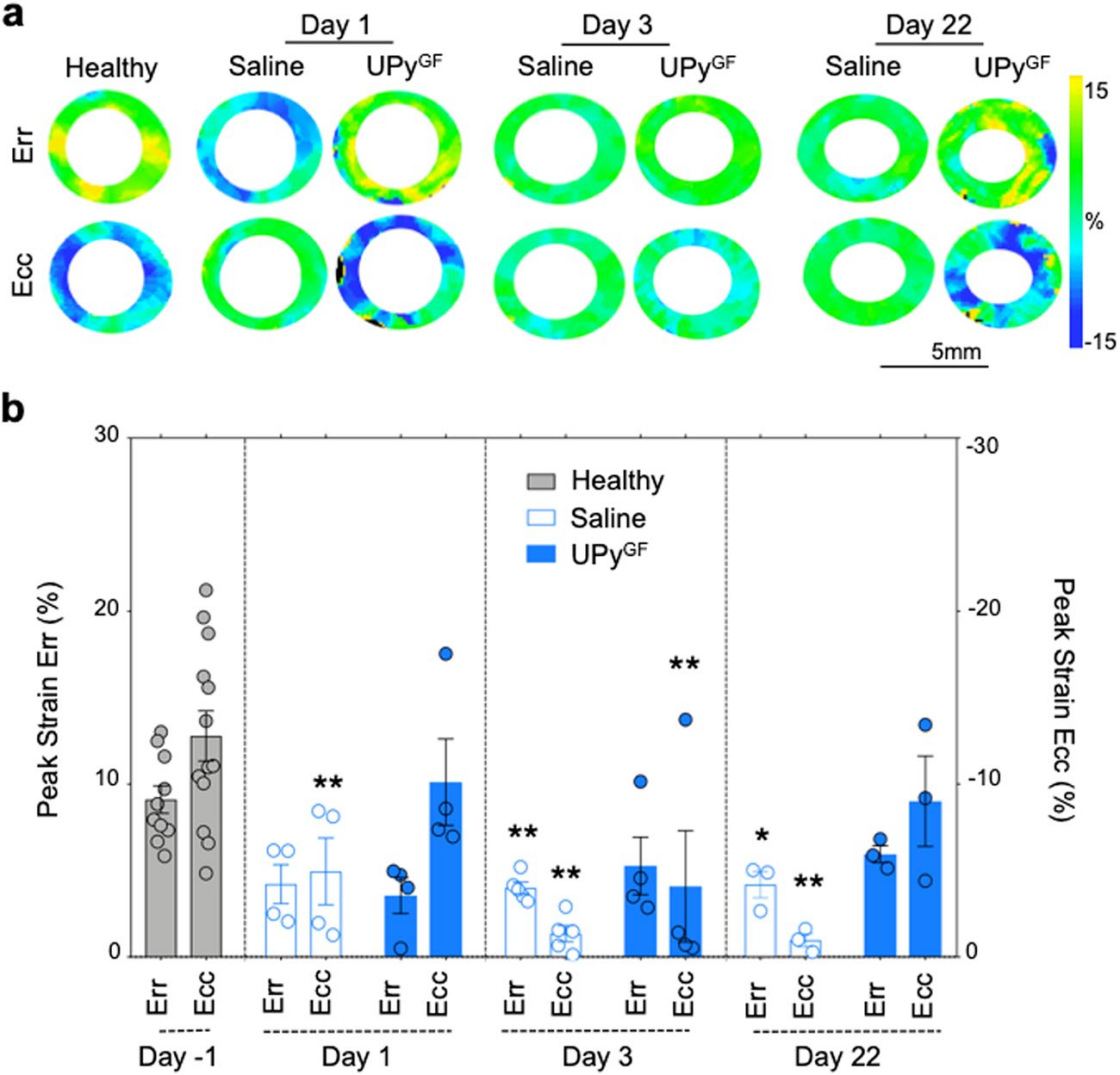
UPy^GF^-hydrogel injection maintains cardiac strain as assessed by serial tagging MRI. (**a**) Longitudinal assessment of circumferential strain (Ecc), and radial strain (Err) for healthy hearts and infarcted hearts on day 1, 3 and 22 after I/R injected with saline or UPy^GF^-hydrogel. (**b**) Ecc and Err values for healthy myocardium and infarcts. Each dot represents an individual mouse; healthy mice (n = 13), saline injected mice (n = 4, day 1; n = 5, day 3; n = 3, day 22), UPy^GF^ injected mice (n = 4, day 1; n = 4, day 3; n = 3, day 22); bars represent mean ± s.e.m.; Mann-Whitney U test; *P < 0.05, **P < 0.01 compared to healthy values.

As the UPy^GF^-hydrogel could possibly affect preservation of regional contractility of the heart, Err and Ecc were longitudinally assessed on day 1, day 3 and day 22 after I/R (Fig. 5a). For saline injected hearts, midventricular peak Err at day 1 (4.2 ± 1.1%), day 3 (4.0 ± 0.3%) and at day 22 (4.2 ± 0.8%) appeared reduced. Yet only at day 3 and day 22 this decrease was significant compared to the Err of healthy myocardium (9.1 ± 0.8%). For the UPy^GF^-hydrogel treated hearts, midventricular peak Err at day 1 (3.5 ± 1.0%), day 3 (5.3 ± 1.7%) and day 22 (5.9 ± 0.5%) appeared not significantly decreased compared to Err of healthy myocardium (Fig. 5a,b), indicating improved regional strain. Furthermore, the Ecc of saline injected hearts was declined at all timepoints after I/R (day 1: −4.3 ± 2.4%; day 3: −1.3 ± 0.5%; day 22: −1.0 ± 0.7%), and significantly different from Ecc of healthy myocardium prior to MI (−12.8 ± 1.4%). For the UPy^GF^-hydrogel treated hearts, Ecc was significantly reduced at day 3 (−4.1 ± 3.2%) compared to Ecc of healthy hearts. Ecc of UPy^GF^-hydrogel injected hearts appeared at day 1 (−10.1 ± 2.5%) and day 22 (−9.0 ± 1.0) after I/R, similar to healthy myocardium prior to MI (Fig. 5a,b; Supplementary Movie 2,3). These data clearly indicated that the UPy^GF^-hydrogel preserved myocardial strain values, suggesting a protective effect on muscular deformation during cardiac remodeling.

### Left ventricular function improves in hydrogel treated heart after MI

**Figure 6:**
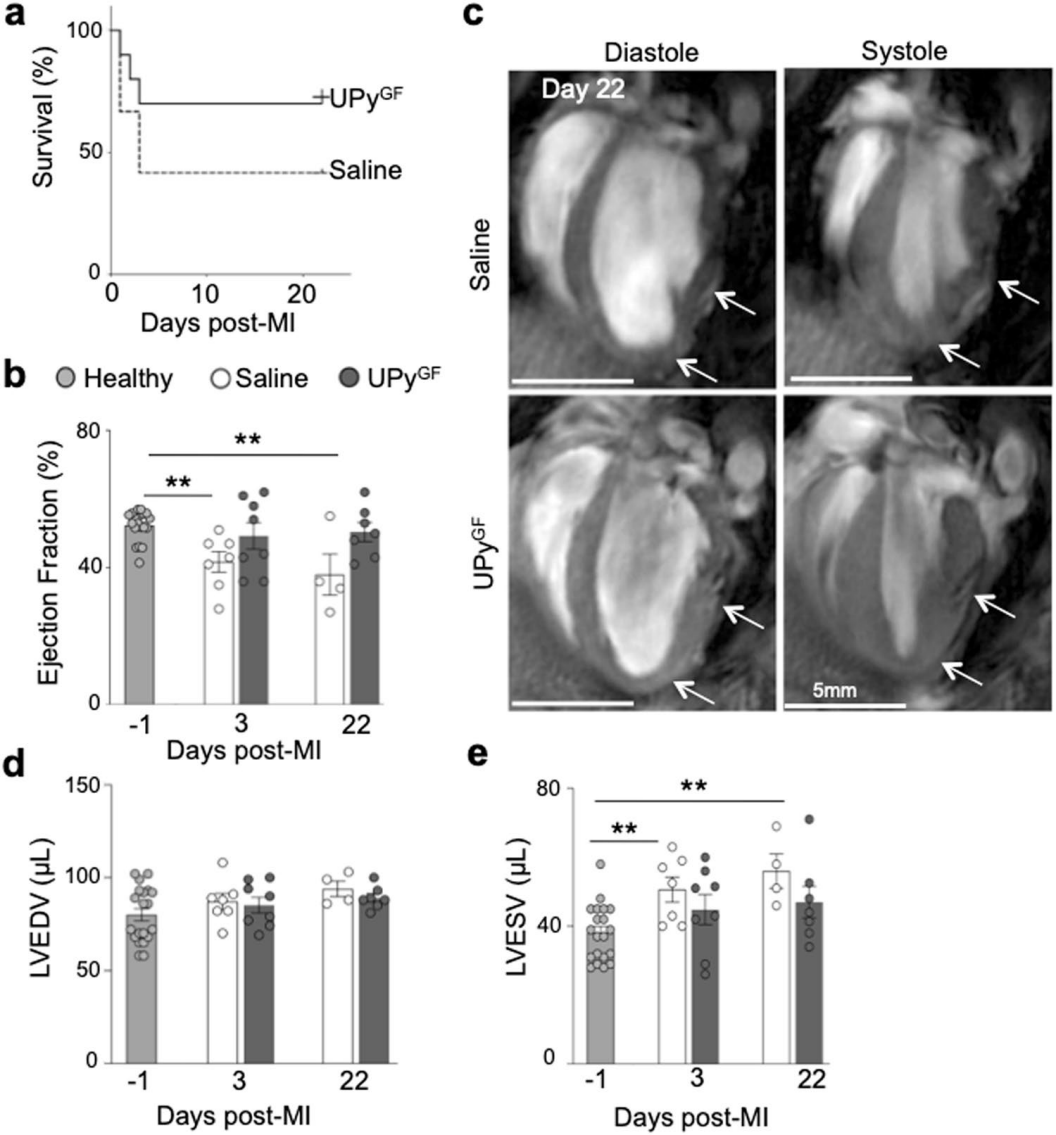
Cardiac cine MRI reveals reduced LV remodeling after I/R injected with UPy^GF^-hydrogel. (**a**) Survival of Saline and UPy^GF^-hydrogel injected mice after ischemia reperfusion injury of the heart (Kaplan-Meier survival curve). (**b**) Ejection fraction. (**c**) End-diastolic and end-systolic long axis MR images of hearts on day 22 after I/R injected with saline or UPy^GF^-hydrogel. (**d**) Left ventricular end-diastolic volume (LVEDV). (**e**) Left ventricular end-systolic volume (LVESV). Each dot represents an individual mouse; healthy mice (n = 20), saline injected mice (n = 7, day 3; n = 4, day 22), UPy^GF^ injected mice (n = 8, day 3; n = 7, day 22); bars represent mean ± s.e.m.; Mann-Whitney U test; **P < 0.01.

Survival rates for the saline and UPy^GF^-hydrogel treated mice were found not significantly different, however a trend towards improved survival in the UPy^GF^-hydrogel treated mice was observed; 77.7% of the initial UPy^GF^-hydrogel treated group survived, while 36.4% of the initial saline treated group survived (Mantel-Cox analysis p = 0.19, Fig. 6a). In addition to overall survival, LV global function was evaluated by calculation of the ejection fraction (EF) from cine MRI. The mean healthy EF was determined from healthy hearts (51.7 ± 1.5%). After I/R, the EF in saline injected hearts appeared significantly reduced after I/R (day 3: 41.7 ± 3.5%; day 22: 38.0 ± 6.0%). In UPy^GF^-hydrogel-treated hearts post-infarction EF remained similar (day 3: 49.3 ± 3.8%; day 22: 50.5 ± 5.1%) to the EF of healthy hearts (Fig. 6b,c). In healthy hearts LV end-diastolic volume (LVEDV; 80.0 ± 3.3 μL) and end-systolic volume (LVESV; 38.1 ± 1.8 μL) were measured. After infarction, the LVEDV in saline injected day 3: 87.1 ± 4.4 μL; day 22: 94.0 ± 8.3 μL) and UPy^GF^-hydrogel-treated (day 3: 85.3 ± 4.2 μL; day 22: 89.1 ± 2.3 μL) hearts appeared not significantly elevated (Fig. 6d). The post-infarction LVESV in saline injected hearts appeared significantly elevated after I/R (day 3: 50.6 ± 3.6 μL; day 22: 56.0 ± 3.6 μL). Yet in UPy^GF^-hydrogel-treated hearts post-infarction LVESV remained similar (day 3: 44.8 ± 4.32 μL; day 22: 46.99 ± 4.6 μL) to the LVESV of healthy hearts (Fig. 6e). We concluded that infarcted hearts treated with UPy^GF^-hydrogel were able to maintain its LV cardiac function after I/R, despite the tissue changes.

### Histology confirms the tissue changes detected by multiparametric cardiac MRI

**Figure 7:**
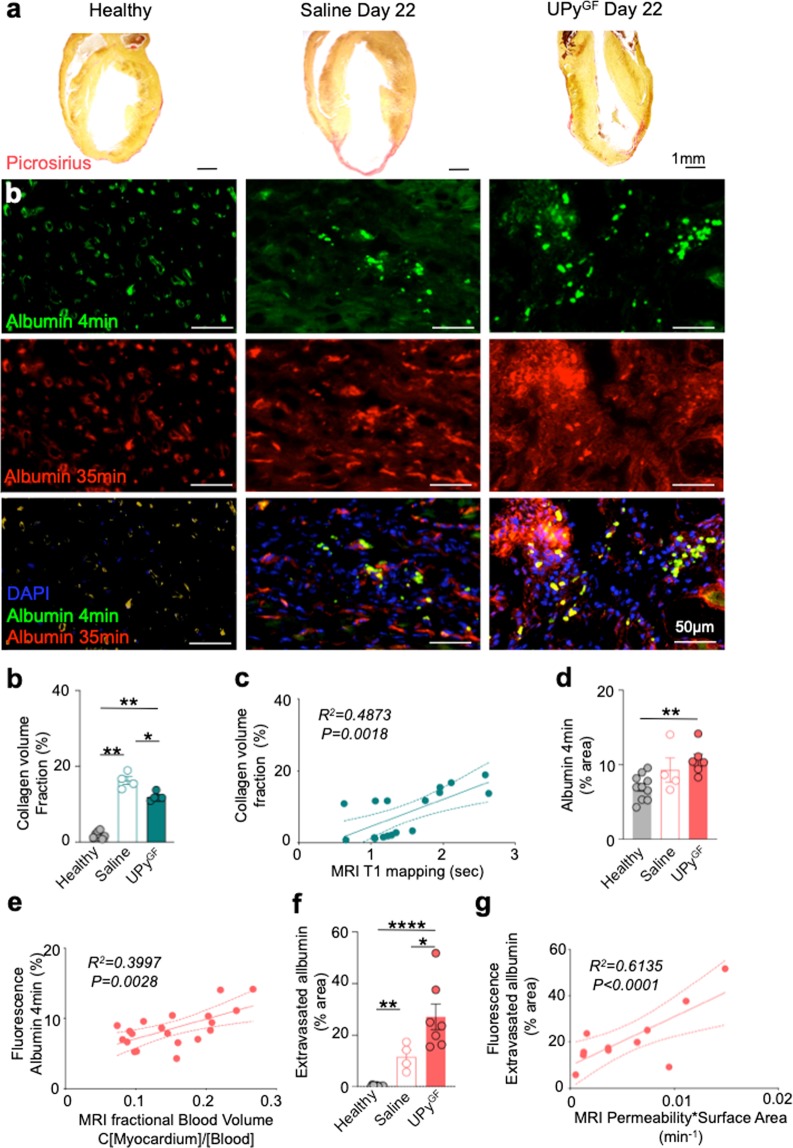
Histological validation of cardiac MRI data for infarcted hearts 22 days after I/R injected with UPy^GF^-hydrogel. (**a**) Representative images of picrosirius red for collagen. (**b**) Green fluorescence staining showing albumin-GFP injected at t = 4 min before euthanasia showing microvascular density and GdDTPA-albumin-RhoB at t = 35 min (after cardiac MRI) displaying vessel permeability in healthy, saline infarcts and UPy^GF^-hydrogel injected infarcts at day 22 after I/R. Merged images with blue: nuclear staining with DAPI, green: albumin-GFP and red: GdDTPA-albumin-RhoB. (**c**) Collagen volume fraction (n = 8 for healthy, n = 4 for day 22 Saline, n = 5 for day 22 UPy^GF^ mice). (**d**) Correlation of histological collagen volume fraction and MR-based T_1_ mapping (*R*^2^ and *P* based on n = 17). (**e**) Albumin t = 4 min showing microvascular density (% area; n = 10 for healthy, n = 4 for day 22 Saline, n = 6 for day 22 UPy^GF^ mice). (**f**) Correlation of fluorescence albumin t = 4 min and MR-based fractional blood volume (*R*^2^ and *P* based on n = 20). (**g**) Extravasated albumin showing permeability (% area; n = 10 for healthy, n = 4 for day 22 Saline, n = 6 for day 22 UPy^GF^ mice). (**h**) Correlation of fluorescence extravasated albumin and MR-based permeability*Surface Area (*R*^2^ and *P* based on n = 20). Each dot represents an individual mouse; bars represent mean ± s.e.m.; One-way ANOVA with Kruskal-Wallis post-hoc test; *P < 0.05, **P < 0.01, ****P < 0.0001.

Microscopic evidence of reduced collagen content by picrosirius red staining is shown in UPy^GF^-hydrogel treated hearts 22 days after I/R (Fig. 7a). Collagen content of each group, namely healthy hearts (1.93 ± 0.7%), saline treated (14.8 ± 1.2%) and UPy^GF^-hydrogel (11.7 ± 0.5%) treated hearts 22 days after I/R, was significantly different (Fig. 7c). Cardiac MRI T_1_ values correlated strongly with values of collagen content (*R*^2^ *=* *0.6980; P* *=* *0.0018)*, confirming T_1_ mapping is a reliable approach to assess myocardial fibrosis after MI (Fig. 7d). Vascular features were assessed in histology by comparing early (FITC-albumin) with late (GdDTPA-albumin-RhoB) albumin localization. Fluorescence microscopy showed enhanced angiogenesis displayed by higher albumin signal at early and late time points in UPy^GF^-hydrogel treated myocardium 22 days postinfarct (Fig. 7b). The area fraction of early albumin, which is a measure for vascular density, was significantly increased for the UPy^GF^-hydrogel treated hearts (10.8 ± 0.8%) compared to healthy (7.4 ± 0.4%) and saline injected (8.9 ± 0.6%) hearts (Fig. 7e). As MR-based fBV is also calculated from an early albumin time point, fBV correlated well with early fluorescent FITC-albumin, (R^2^ = 0.4242, P < 0.0001; Fig. 7f). Subtraction of the early FITC-albumin area from the late extravasated GdDTPA-albumin-RhoB revealed the amount of extravasated albumin. For healthy myocardium only 0.5 ± 0.1% extravasated. The % area extravasated albumin for saline treated myocardium (11.5 ± 1.4%) was significantly higher compared to extravasation in healthy myocardium. Extravasation of albumin in UPy^GF^-hydrogel treated myocardium (23.3 ± 3.7%) was significantly increased compared with extravasation in healthy and saline injected myocardium (Fig. 7g). These histology-based findings were in positive correlation with the MR-based PS (R^2^ = 0.5751, P < 0.0001). To conclude, these findings confirmed the MR-based results for day 22, displaying reduced fibrosis and enhanced revascularization in the UPy^GF^-hydrogel treated group.

## Discussion

**Figure 8:**
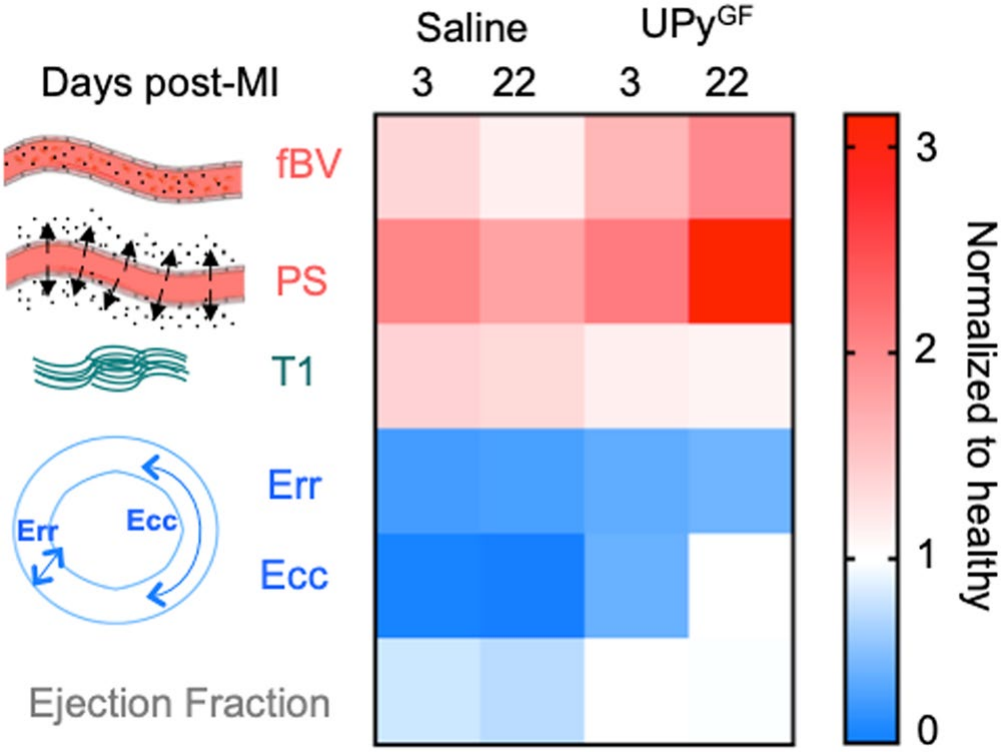
Longitudinal multiparametric cardiac MRI comparing saline and UPy^GF^-hydrogel injected myocardial healing. Sequential tissue changes by saline and UPy^GF^-hydrogel injections to the infarcted hearts at 3 and 22 days after I/R comparing injection normalized to mean values of healthy myocardium. (fBV: fractional blood volume; PS: permeability*surface area product; T_1_: T_1_ relaxation time; Err: radial strain; Ecc: circumferential strain).

In this study, we describe two advances: (1) a noninvasive multiparametric cardiac MRI tool aimed to characterize key regenerative processes after I/R injury; we established a unique triple-marker MRI approach assessing microvascular features, fibrosis and regional muscle strain at different time points of cardiac healing following hydrogel delivery. (2) Our noninvasive multiparametric MRI approach demonstrated the therapeutic effects of a VEGF/IGF1-loaded UPy^GF^-hydrogel after I/R injury by highlighting (i) the enhanced angiogenesis by fractional blood volume (fBV) and permeability * surface area product (PS), (ii) improved resolution of fibrosis by T_1_-mapping and (iii) elevated mechanical support as well as (re)muscularization by strain analysis of infarcted murine hearts (Fig. 8). Both claims are supported by gold standard *ex vivo* immunohistochemical analysis displaying an excellent correlation of angiogenic and fibrotic MRI findings, showing the potential of this translational triple-marker MRI approach.

As the search for a novel and effective therapy to prevent the progression of post-MI heart failure continues, we tested a supramolecular UPy^GF^-hydrogel loaded with VEGF/IGF1. Optimally, time-to-treatment should be at the earliest possible time after ischemic injury. The injection of the UPy^GF^-hydrogel before reperfusion was intended to prevent damage from the oxidative stress of the reperfusion injury with release of oxygen-related free radicals^29–31^. The UPy^GF^-hydrogel prevented initial damage by providing a mechanical support in the tissue after injection that protects the injured myocardium in a high shear environment^14^. Furthermore, growth factor release from the UPy^GF^-hydrogel peaked at day 2–3 after injection (Fig. 1a), which is an ideal timing to initiate angiogenesis, as the revascularization is initiated at day 3 after I/R injury^29,31^. Despite our promising preclinical results and the fact that the delivery at time of reperfusion seems clinically optimal, future studies should examine the optimal time frame for treatment, both in clinical outcomes and patient burden.

Although stem cell therapy for cardiac repair and regeneration holds promising therapeutic potential, its beneficial effect has been greatly attributed to the release of growth factors, such as VEGF and IGF1, acting in a paracrine fashion on resident cells^4,32^. IGF1 has been extensively studied to treat the heart after MI as it counteracts adverse cardiac remodeling working directly on cardiomyocytes and inflammatory cells; reduced apoptosis^33,34^, cardioprotective effects against oxidative stress-dependent cell death^35^ and modulation of the inflammatory response^8^ at the infarcted area are possible underlying mechanisms. IGF1 works through receptor binding and intracellularly activates the protein kinase B pathway, stimulating cell growth and proliferation, and inhibiting apoptosis^6–8^.

VEGF, a cytokine that initiates the formation of new vessels, has been broadly examined as a treatment option to prevent heart failure after MI, as it also exerts cytoprotection, tissue regeneration, and neurohormonal effects^5^. VEGF works through receptor activation and molecular mediators resulting in inhibition of apoptosis by caspase-9 phosphorylation, vasodilation promoted through eNOS (endothelial nitric oxide synthase) activation with nitric oxide production, and activation of kinases inducing cellular proliferation, adhesion and migration. *In vivo* evidence has suggested that VEGF works both on local cells of the cardiac niche (e.i. stromal cells), as well as on the recruitment of distant bone marrow-derived cells (e.i. hematopoietic stem cells, monocytes), starting from cellular release, followed by cell migration, adhesion and, finally, diapedesis towards the damaged tissue^5^.

One of the major limitations for the therapeutic use of both VEGF and IGF1 is the very short half life following injection^5,36^. Entrapment of these growth factors in an engineered degradable hydrogel overcomes this limitation enabling prolonged release of VEGF and IGF1 over 7 days during the phase of active cardiac healing^3^. The burst release of VEGF was slower than IGF1. Previously published data have shown similar burst release for VEGF^37^ and IGF1^14^. However, the reason for discrepancy in burst release between VEGF and IGF1 has not been further explored. Possible explanations are partial degradation of VEGF hindering ELISA detection^38^ or difference in size between VEGF (39 kDa) and IGF-1 (7.6 kDa)^39^. The correct intramyocardial injection was imaged by incorporating USPIOs to the UPy^GF^-hydrogel. The very short T_2_* of the injected USPIOs created a susceptibility artifact, slightly overestimating the injected hydrogel area. Because of this, the hearts were isolated and placed in a tube, so the effective intramyocardial placement of the engineered hydrogel could be verified. These *in vitro* and *ex vivo* data demonstrated feasibility of the UPy^GF^-hydrogel as an intramyocardial carrier system in murine hearts. For porcine hearts, minimally invasive delivery of a UPy-hydrogel loaded with Gd through a catheter has been shown, emphasizing the translational potential of the gel^15^.

The continuous search to lower mortality after MI also demands methods to assess the risks of developing adverse cardiovascular events. Risk stratification is crucial for decision-making in existing treatment options, such as revascularization, as well as robustly evaluating novel regenerative therapies to prevent progression to post-MI heart failure. LV ejection fraction (≤30%) has become a primary method for stratifying risk like anticipating sudden cardiac death^40^. Yet, even patients with LV EF >30% may still suffer sudden cardiac death and this method does not provide any insights on aspects of cardiac healing^40^. The evaluation of myocardial tissue changes post-MI can deliver a patient-specific risk stratification, ultimately contributing to personalized medicine.

Cardiac MRI has emerged as a leading imaging modality assessing various components of post-MI myocardium for risk assessment and personalized therapeutic decision making. Until now, measuring infarct size by LGE MRI has been a very strong predictor of mortality and heart failure^41^. Our study revealed that both the saline and UPy^GF^-hydrogel treated hearts had similar infarct size upon LGE MRI at day 1 after MI, meaning similar initial risk for developing adverse cardiovascular events. A mouse model of I/R injury with small infarct sizes was used to mimic clinical conditions^28,42^.

Cardiovascular albumin-based DCE MRI is a promising method to evaluate functional vascular features such as vascular density and permeability in mice^19,20^ and man^43,44^. Serial imaging showed that UPy^GF^-hydrogel therapy elevated the infarct fBV, a measure for microvascular density at day 3 and day 22 after I/R injury. Vascular permeability (PS) was not significantly different at day 3 post-MI. As permeability early after MI correlated with leukocyte transmigration, this indicated indirectly that the inflammatory reaction was not enhanced early post-MI^20^. PS appeared only significant in UPy^GF^-hydrogel treated mice late (day 22) after I/R injury. As permeability is a major indicator for angiogenesis^45^, this may indicate that angiogenesis was still ongoing at day 22 post-MI in the infarcted myocardium by prolonged release of VEGF from the UPy^GF^-hydrogel.

Furthermore, MR-based T_1_-mapping assessing fibrosis has been shown to be a promising method for risk evaluation both preclinically^24,46^ and clinically^21,22^. Our results showed that intramyocardial injection of UPy^GF^-hydrogel alleviated myocardial fibrosis at day 3 and day 22 post-MI. These findings were confirmed by histomorphometric analysis.

Global strain has recently been shown to have incremental prognostic value for mortality superior to LV ejection fraction and infarct size^47,48^. To include both intramycardial injection sites of saline or UPy^GF^-hydrogel, we chose to assess midventricular Ecc and Err strain serially by tagging MRI^23^. However, the use of tagging MRI for strain assessment resulted in the exclusion of multiple later timepoints due to tagging fading^49^. Other strain assessment approaches, such as feature tracking, might result in less exclusion of images, but have a risk of unrealistic results with local stain variations as we expected in MI^23^. Therefore tagging MRI is still the gold standard to rely on. We found compromised strain values for the saline injected group, whereas the strain values of the UPy^GF^-hydrogel treated group remained similar to healthy strain values. This is likely due to the initial mechanical support of the UPy-hydrogel combined with the sustained release of growth factors to enhance survival of cardiomyocytes^34^.

In addition to classical MR assessment of infarct size by LGE MRI and LV global function by cine MRI postinfarct, each separate MRI method, i.e. DCE MRI^19^, T_1_ mapping^22^ and tagging MRI^23^, has proven potential additional value for assessing distinct biomarkers of risk prediction after MI. Yet this combined triple-marker MR imaging method gives clear insights on post-MI tissue changes in one imaging session, allowing spatial and temporal comparison of the therapeutic effects of the UPy^GF^-hydrogel. Other multi-biomarker studies have included simultaneous measurement of multiple tissue properties in a single acquisition measuring T_1_, T_2_ and T_2_*^50,51^. However, these measurements cannot provide functional data on regional muscle deformation or endothelial barrier function of the heart. Some studies have used cardiac positron emission tomography (PET)/MRI to assess cardiac inflammatory reactions, in addition to standard LGE, T_1_ and T_2_ MRI measurements^52^. Although PET is highly sensitive, for human applications MRI is favored as it is more widely available, nonradioactive, independent of cyclotron access, and cheaper.

This serial cardiac MRI strategy achieved (i) 3D characterization of vascular features showing increased microvascular density (fBV) and vascular permeability (PS) by DCE-MRI, (ii) MR-based evaluation of myocardial fibrosis picturing improved T_1_ values of fibrosis and (iii) MR-based radial and circumferential strain analysis displaying enhanced regional wall motion in UPy^GF^-hydrogel treated infarcts. Improved global LV function was attributed to increased angiogenesis, enhanced resolution of fibrosis and elevated mechanical support as well as (re)muscularization in infarcted hearts treated with UPy^GF^-hydrogel. We envision that such multiparametric platforms will enable simultaneous studies of hallmarks for cardiac regeneration, at high resolution, to improve insights into the efficacy of novel regenerative cardiac therapies. Ultimately, this integrated *in vivo* MRI tool, can be translated clinically to provide longitudinal information of the regenerative aspects of novel cardiac therapies after MI.

## Methods

### Statement

All experiments and methods were performed in accordance with relevant guidelines and regulations. All mouse procedures were approved by Maastricht University Animal Care Committee, The Netherlands (DEC2014–053).

### Synthesis and characteristics of UPy^GF^-hydrogel

Based on the fourfold hydrogen bonding supramolecular ureido-pyrimidinone units were coupled with alkyl-urea to 10k poly(ethylene glycol) to synthesize the UPy-hydrogel (SyMO-Chem BV, Eindhoven, The Netherlands)^14^. A 10 wt% UPy-hydrogel was made by dissolving 10 wt% UPy-PEG in 90 wt% saline to a final pH 9. After one hour of stirring, the liquefied UPy-hydrogel was mixed with mouse recombinant VEGF and IGF1 (V4512–5UG and I8779–50UG, Sigma-Aldrich, St Louis, MO, USA) to a concentration of 500 ng/ml of both GF. In addition, a second batch of the UPy^GF^-hydrogel was mixed with 13,6 μg/ml USPIOS (Sinerem, Guerbet, Villepoint, France) for *in vivo* tracking after delivery. To assess the cumulative release of IGF1 and VEGF, 100 μl of the liquefied UPy-hydrogel was placed in a pipette at 24-well plate millicell hanging culture inserts (PIEP12R48, polyethylene terephtalate, 8.0μm; Merck Millipore, Darmstadt, Germany) and solidified by decreasing to pH 6.5 while adding hydrochloric acid. The MilliPores where surrounded with 800 μl phosphate-buffers saline (PBS) and placed at a rotational shaker (100 rpm) at 37 °C for 7 days. The PBS surrounding the inserts was refreshed daily and the collected supernatant was quantified for VEGF and IGF1 by enzyme-linked immunosorbent assay detection (Mouse IGF1 ELISA Kit and Mouse VEGF ELISA Kit, Sigma-Aldrich, St Louis, USA).

### Mice

Male outbred 12 ± 1weeks old OF1 mice (n = 35) were purchased from Charles River. A first batch of n = 14 healthy mice were used for implementation of cardiac MRI, and subsequent histological analysis. A second batch of n = 20 healthy mice were imaged prior to infarct induction and were randomly assigned to the saline injected (n = 11) or UPy^GF^-hydrogel injected (n = 9) group during I/R injury for serial cardiac MRI and histology at day 22 post infarction. To localize USPIO-loaded UPy^GF^-hydrogel after I/R injury by *ex vivo* MRI n = 1 mouse was used as a proof-of-concept. For induction of I/R surgery and *in vivo* cardiac MRI, anesthesia of the mice was induced with 4% isoflurane in 0.3 L/min oxygen and maintained with 1–2% isoflurane. During anesthesia, the body temperature was monitored with rectal thermometry and maintained at 35.5 ± 0.5 °C using a heating system. All mouse procedures were approved by Maastricht University Animal Care Committee, Netherlands.

### Myocardial I/R injury model

A well-established left anterior descending (LAD) coronary artery ligation model was used^26,42^. In short, mice were anesthetized, endotracheally intubated and mechanically ventilated (Harvard Apparatus). Surgery was performed under an Olympus microscope. After thoracotomy, a 30 min ligation of the LAD coronary artery was performed with a 8–0 monofilament suture and a PE-10 tube placed between the LAD coronary artery and the suture to protect the vessel from injury. Although there is still some controversy about the ideal timing of injection after MI, we have well considered several options taking into account the nature of our regenerative therapy and its dynamics. The most favorable time-to-treatment should be as early as possible after ischemic injury to prevent damage^29–31^. Two minutes prior to reperfusion, two intramyocardial injections of 10 μl saline or 10 μl UPy^GF^-hydrogel were placed on both sides of the ischemic area (Fig. 1c) by a 90 degrees curved needle (0.3 mL U-100 insulin syringe, 29-gauge, BD Bioscience). At the end of the ischemic period, the LAD coronary artery ligation was removed and reperfusion of the LV was visually verified. Before and every 12 h until 72 h after the surgery, mice received buprenophine (0.05 mg/kg, SC) injections as analgesia. During the first 24 h after surgery, mice were kept in a recovery unit at 28 °C.

### Cardiac MRI acquisition

A 9.4 T horizontal MRI scanner with 72-mm-diameter volume transmit coil with a four-element mouse cardiac phased-array surface receive coil (Bruker, Ettlingen, Germany) was used to perform all MRI experiments.

#### *Ex vivo* MRI protocol

Hearts were harvested 30 min after I/R injury and isolated to verify the intramyocardial localization of USPIO-loaded UPy^GF^-hydrogel. The pericardium was removed and the hearts were fixated in paraformaldehyde (polyoxymethylene, P6148, Sigma-Aldrich, St Louis, USA) in an Eppendorf tube. This tube was positioned in the isocenter of the magnet and imaged by T_2_*-weighted imaging performed with a standard gradient echo sequence using a thickness of 0.5 mm in long axis (20 slices) and short axis (25 slices) covering the whole heart. Following parameters were used: TE = 7 ms; flip angle = 30°; TR = 3355 ms; TEacq = 10.9 ms; spectral width = 407 Hz; FOV_long axis_ = 40 × 40 mm; FOV_short axis_ 30 × 30 mm^2^; N_x_ × N_y_ = 192 × 192 with zero filling to 256 × 256; acquisition time = 10m44s232ms.

#### *In vivo* MRI protocol

ECG electrodes were placed at the front paws to monitor the heart rate, which was maintained at 400–600 bpm by adjusting the isoflurane level. A respiratory sensor balloon was placed on the abdomen and the respiratory rate was kept at 70 ± 10 bpm (SA Instruments Model 1025, Stony Brook, NY, USA). The mouse was placed in the magnet so that the heart was positioned at the isocenter of the magnet in the middle of the phased array coil. Retrospective triggering was used to distinguish cardiac and respiratory phases from the images after acquisition. For healthy baseline and mice at days 3 and 21 after I/R, one imaging session accounted for DCE, T_1_-mapping, tagging and volumetric Cardiac MRI (5–7 days prior to I/R surgery). For day 1 after I/R injury, the imaging session was minimized to only tagging and LGE MRI, to diminish the time under anesthesia and to consequently optimize survival. At day 3 and 22 after I/R, DCE, T_1_-mapping, tagging and volumetric MRI were performed again (Fig. 1e).

#### Cardiac DCE MRI with precontrast T_1_-mapping

Prior to administration of the contrast agent, T_1_-weighted stacks of 2D fast low‐angle shot (FLASH) images covering the whole heart were acquired with a series of variable flip angles (2°, 5°, 8°, 11°, 13°) to determine the endogenous precontrast T_1_ as described in previous research^19,24^. In short, a retrospectively triggered FLASH sequence with constant TR was used with variable flip angles. An indwelling tail catheter was used to intravenously inject 150 μl (100 mg/ml) macromolecular bovine serum albumin labeled with gadolinium diethylenetriaminepentacetate (GdDTPA) and rhodamine B (GdDTPA-albumin-RhoB, 82 kDa, SyMo-Chem, Eindhoven, The Netherlands) at a rate of 50 μl/min^19^. Immediately after administration of the contrast agent a dynamic series of images was acquired with a flip angle of 13° to determine post contrast T_1_ over time for quantification of contrast accumulation. Other imaging parameters were: TR = 10 ms; TE = 1.784 ms; n of repetitions = 22; FOV = 30 × 30; slice thickness = 10 mm; spectral bandwidth = 100,000 Hz; N_x_ × N_y_ = 128 × 64 with zerofilling to 128 × 128 ; number of slices = 15; cardiac frames = 6; acquisition time = 4m1s200ms.

#### Tagging MRI

Midventricular tagged MRI images were obtained by preceding a 2D-FLASH sequence with a SPAMM preparation step applying a tagging grid with following parameters: distance = 0.5 mm; thickness = 0.25 mm; angle = 45°; delay = 1.4 ms. These short axis tagged MRI images were positioned between the base and apex of the heart, containing the UPy^GF^-hydrogel or saline injection site. This sequence was prospectively gated and respiratory triggered per phase step with a trigger delay of 1 ms. The tagging was applied prior to the systolic phase and acquired images were ordered from the systolic to the diastolic phase (Supplementary Movie 1). Other imaging parameters were: α = 13°; TR = 15 ms; TE = 2.549 ms; FOV = 30 × 30 mm; N_x_ × N_y_ = 256 × 128 with zerofilling to 256 × 256; cardiac frames = 16; acquisition time depends on cardiac triggering efficiency. Tagging series of insufficient image quality to assess tagging grid deformations^49^ because of tagging grid fading were excluded from analysis.

#### Cardiac Volumetric MRI

A retrospectively gated FLASH sequence (Intragate, Bruker, Ettingen, Germany) was applied to acquire cine images of the long axis orientation and determine the position of the short axis DCE MRI or the LGE MRI. The following parameters were used: α = 10°; TR = 8 ms; TE = 3 ms; n of repetitions = 200; FOV = 30 × 30 mm; N_x_ × N_y_ = 192 × 192 with zerofilling to 256 × 256; slice thickness = 1 mm; cardiac frames = 15; acquisition time = 5m9s200ms.

#### LGE MRI

One day after surgery the infarct size was determined by LGE MRI with an increased flip angle preparation step before a T_1_-weigthed 3D-FLASH sequence. The LGE MRI images were acquired 20 min after 100 μl gadoterol (GdDTPA, 0.015 mmol/kg, Prohance, Bracco Diagnostics Inc.) was injected intravenously via an indwelling tail vein catheter at the same rate as above. The applied imaging parameters were: α = 30°; TR = 10 ms; TE = 1.784 ms; n of repetitions = 50; FOV = 30 × 30; slice thickeness = 10 mm; spectral width = 100,000 Hz; N_x_ × N_y_ = 128 × 64 with zerofilling to 128 × 128; number of slices = 15; cardiac frames = 6; acquisition time = 8m30s0ms.

### Cardiac MRI data analysis

#### T_1_, fBV and PS

Cardiac T_1_-mapping and DCE MRI data analysis was done with Matlab (MathWorks Inc., USA). GdDTPA-albumin-RhoB concentrations were derived from the cardiac DCE MRI data on a pixel-by-pixel basis^19,24^. In short, mean myocardial R_1_ values (R_1_pre; R_1_ = 1/T_1_) Eq. 1:

1 $$I=\frac{{M}_{0}\,\sin \,\alpha (1-{e}^{-TR\cdot R{1}_{pre}})}{1-\,\cos \,\alpha \cdot {e}^{-TR\cdot R{1}_{pre}}}$$

where *I* is the signal intensity as a function of the pulse flip angle α, TR is the repetition time and the pre‐exponent term M_0_ includes the spin density and T_2_ relaxation effects, assumed to be unaffected by the contrast agent. Postcontrast R_1_ values were calculated from signal intensities precontrast and postcontrast (*I*_pre_ and *I*_post_; Eq. 2):

2 $$\frac{{I}_{{\rm{pre}}}}{{I}_{{\rm{post}}}}=\frac{{M}_{0}\,\sin \,\alpha (1-{e}^{-TR\cdot R{1}_{pre}})/(1-\,\cos \,\alpha \cdot {e}^{-TR\cdot R{1}_{pre}})}{{M}_{0}\,\sin \,\alpha (1-{e}^{-TR\cdot R{1}_{post}})/(1-\,\cos \,\alpha \cdot {e}^{-TR\cdot R{1}_{post}})}$$

Lastly, concentrations of the contrast agent were derived from the measured *relaxivity* (*R*) of GdDTPA-albumin-RhoB (*R* = 130 mM^−1^s^−1^ at 9.4 T; Eq. 3):

3 $$[GdDTPA-albumin-RhoB]=\frac{1}{R}({\rm{R}}1{\rm{post}}-{\rm{R}}1{\rm{pre}})$$

Regions of interest (ROIs) for T_1_, fBV and PS were defined as healthy before I/R injury for the entire myocardium and as infarcted for a hyper intense infarct region defined by LGE MRI images. From the temporal changes in these normalized concentrations fBV and PS were quantified. First, fBV was derived from the extrapolation of a linear regression of the normalized concentrations to the time of administration of the contrast. Second, the slope of these normalized concentration values (Fig. 1b), the rate of normalized concentration increase or PS (min^−1^), was derived from a linear regression of the normalized concentrations over time. The PS quantified with GdDTPA-albumin-RhoB exhibited the extravasation of albumin from blood vessels and its accumulation in the tissue. Cardiac parametric T_1_, fBV and PS maps were projected to indicate the mean value in each pixel.

#### Strain

Strain calculations were done by using Wolfram Mathematica v10 based software, adapted from previous research^25^. The method obtained the circumferential (Ecc) and radial (Err) strains from the tagging MRI data (Fig. 2e–g) and is based on local tagging frequency estimations described elsewhere^25,53^. Briefly, the Gabor transform was applied to construct a local frequency representation of the tagging images A Gaussian filter was used to calculate the Gabor transform in each pixel and a single spatial frequency covector ω_t_ was extracted (t is the time frame of the MRI acquisition the covector was calculated from). Each ω_t_ is related to its counterpart in the first frame ω_0_ for the same material point through the deformation tensor F Eq. 4):

4 $${\omega }_{{\rm{t}}}={\omega }_{0}{{\rm{F}}}^{-1}$$

Where ω_t_ and ω_0_ are are the frequencies of corresponding material points at different moments in time and F is the deformation tensor (Eq. 5). It is assumed that at the moment of applying the tagging grid t_0_, which is before acquisition of the image, the tag frequency is known and and equal for all material points. To be able to solve for F, frequency-matrices Ω_t_ and Ω_0_ are derived by combining frequency covectors ω_t_ and ω_0_ for multiple tagging directions extracted tagged images:

5 $$F={({\Omega }_{t}^{T}{\Omega }_{t})}^{-1}{\Omega }_{t}^{T}{\Omega }_{0}$$

Here Ω_T_ is the transpose of Ω. From this the Lagrangian strain tensor (Eq. 6) is defined as followed:

6 $$E=\frac{1}{2}({F}^{T}F-I)$$

Where *I* is an unit tensor. From the 2 × 2-tensor E the Err and Ecc strains can be extracted. Prior to these strain calculations the endocardial and epicardial borders were manually drawn into the image slices to limit the deformation analysis to that area. The peak Err and Ecc were calculated in the whole LV (Fig. 2f) and were analyzed by dividing the LV into 12 segments in the circumferential direction and two in the radial direction, which is a refinement of the American Heart Association (AHA) model. The peak strains of the infarct area segments were compared between the saline and UPy^GF^-hydrogel groups. For healthy controls the same segments were used. For regional strain analysis, the accuracy of tagging MRI is known to be dependent on the image quality^54^. Our data suffered from tag fading, especially in the diastolic phase, which is a known problem for this technique^49^ making it impossible to accurately analyze some datasets. Datasets with excessive tagging grid fading were excluded (Supplementary Fig. 1), which explains the lower number of animals for regional strain analysis than T_1_, fBV and PS analysis.

#### Infarct size, EF

To gain insights into the effect of the UPy^GF^-hydrogel therapy in the heart, infarct size and global LV function were calculated with Segment v1.9 R3819 software (http://segment.heiberg.se). At day 1 postinfarction, 3D LGE MRI images were used to determine infarct sizes of the different saline and UPy^GF^-hydrogel injected LVs. The infarct areas were defined from pixels with a signal intensity of more than 3 standard deviations above the mean signal intensity of the remote area (Fig. 3a). The precontrast 3D FLASH images with 13° flip angle from the healthy control and day 3 and 22 for the saline and UPy^GF^-hydrogel groups were used to determine the LV EF. Segment v1.9 R3819 software was used to manually draw the endocardial and epicardial borders in the short axis images and automatically calculated the EF from the difference in LVEDV and LVESV divided by the LVEDV.

### Histological analysis

#### *Ex vivo* histology preparation

After the MRI experiment (healthy and at day 22 after infarction), 150 μl fluorescein isothiocyanate labeled BSA (FITC-albumin, 100 mg/ml) was intravenously injected via the indwelling catheter as an early albumin marker to confirm MR-based fBV. Mice were sacrificed 4 min after FITC-albumin and 35 min after GdDTPA-albumin-RhoB injection by cervical dislocation while anesthetized. The hearts were isolated, rinsed and fixed in paraformaldehyde. Afterwards, the hearts were mounted on OCT embedding compound (Fisher Scientific, Hampton, NH, USA) and frozen at −80 °C. Hearts were cryosectioned at 7 μm through the short axis at −35 °C.

#### Collagen content

For Picrosirius Red staining of collagen, 3–4 sections per heart were incubated with 0.1% Sirius Red (Direct Red 80, Sigma-Aldrich, St Louis, USA). Sections were visualized with identical exposure settings in a light microscope (3DHistech microscope, Budapest, Hungary). The interstitial collagen fraction (dark red) was determined by quantitative morphometry of the picrosirius-stained sections with with ImageJ software. The density of labeled areas was measured from 3 randomly selected fields of each section at a x10 magnification. The expressed value was the ratio of the dark red collagen area to total area.

#### Fluorescent albumin quantification

In adjacent section, nuclei where stained in blue with 4′,6-diamidino-2-phenylindole (DAPI, D9542, Sigma-Aldrich, St Louis, USA). To visualize fluorescence of FITC-albumin injected for 4 min, GdDTPA-albumin-RhoB injected for 35 min, and DAPI, 3–4 slices were imaged with identical exposure times (3DHistech microscope, CMOS camera). The density of labeled areas was qualitatively estimated from 3 randomly selected fields of each section at a x20 magnification. The % area of FITC-albumin (4 min) was the ratio of the green fluorescence area to total area. Subtraction of this fraction from the red fluorescent fraction (GdDTPA-albumin-RhoB; 35 min injected) provided the contrast leakage from the vasculature and therefore an estimation for the permeability. All fluorescent images were analyzed with imageJ software.

### Statistics

Statistical analysis was performed using GraphPad Prism (version 8.00; GraphPad, San Diego, CA). Normality was checked by the Shapiro–Wilk test. Differences between not normal distributed groups were analyzed for statistical significance with the parametric Mann-Whitney U test. Multiple comparisons were corrected for statistical significance with the Bonferroni-Dunn method. For histology, three groups were compared using a one-way ANOVA with Kruskal-Wallis post-hoc test. To assess correlation between two variables, Pearson’s R^2^ and two-tailed P value were computed. Data are presented as mean ± SEM and a *P* value of ≤0.05 was considered significant.

## Supplementary information

Supplementary information

Supplementary movie 1

Supplementary movie 2

Supplementary movie 3
