# Publisher Correction: Triple-marker cardiac MRI detects sequential tissue changes of healing myocardium after a hydrogel-based therapy

**DOI:** 10.1038/s41598-020-59864-w

**Published:** 2020-02-21

**Authors:** Maaike van den Boomen, Hanne B. Kause, Hans C. van Assen, Patricia Y. W. Dankers, Carlijn V. C. Bouten, Katrien Vandoorne

**Affiliations:** 1 Department of Biomedical Engineering, Cell-Matrix Interaction for Cardiovascular Tissue Regeneration, Eindhoven University of Technology, Eindhoven, The Netherlands; 2 Department of Radiology, University Medical Center Groningen, University of Groningen, Groningen, Netherlands; 3 Department of Radiology, Athinoula A. Martinos Center for Biomedical Imaging, Massachusetts General Hospital, Harvard Medical School, Charlestown, MA United States; 4 Department of Electrical Engineering, Eindhoven University of Technology, Eindhoven, The Netherlands; 5 Institute for Complex Molecular Systems (ICMS), Eindhoven University of Technology, Eindhoven, The Netherlands; 6 Department of Biomedical Engineering, Laboratory of Chemical Biology, Eindhoven University of Technology, Eindhoven, The Netherlands

Correction to: *Scientific Reports* 10.1038/s41598-019-55864-7, published online 18 December 2019

In the original version of this Article, the authors Maaike van den Boomen and Hans C. van Assen were incorrectly indexed. These errors have now been corrected.

