# Supplementary material for: Triple-marker cardiac MRI detects sequential tissue changes of healing myocardium after a hydrogel-based therapy
Source: Sci Rep. 2019 Dec 18;9:19366. doi: 10.1038/s41598-019-55864-7 (PMC6920418; doi:10.1038/s41598-019-55864-7)
Supplement: Supplementary file 1 — Supplementary information [file 41598_2019_55864_MOESM1_ESM.pdf]

Triple-marker cardiac MRI detects sequential tissue changes of healing myocardium after a hydrogel-based therapy

Maaike van den Boomen[1,2,3], Hanne B. Kause[4], Hans C. van Assen[4], Patricia Y.W. Dankers[1,5,6], Carlijn V.C. Bouten[1,5], Katrien Vandoorne[1]

[1] Department of Biomedical Engineering, Cell-Matrix Interaction for Cardiovascular Tissue Regeneration, Eindhoven University of Technology, The Netherlands
[2] Department of Radiology, University Medical Center Groningen, University of Groningen, Groningen, Netherlands.
[3] Department of Radiology, Athinoula A. Martinos Center for Biomedical Imaging, Massachusetts General Hospital, Harvard Medical School, Charlestown, MA, United States.
[4] Department of Electrical Engineering, Eindhoven University of Technology, The Netherlands
[5] Institute for Complex Molecular Systems (ICMS), Eindhoven University of Technology, The Netherlands
[6]Department of Biomedical Engineering, Laboratory of Chemical Biology, Eindhoven University of Technology, The Netherlands

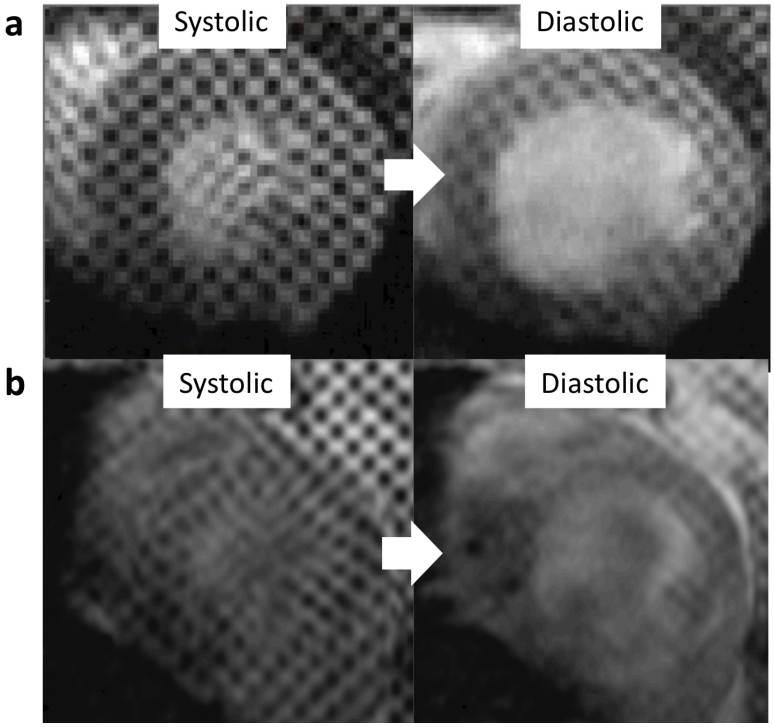

Supplementary Figure 1| **Tag fading in tagging MRI. a,** Representative tagging MR images during systole and diastole with sufficient detail for strain analysis. **b,** Representative tagging MR images during systole and diastole with tag fading happening during irregular heart beats after infarction.

Supplementary movie 1. Cine movie of tagging cardiac magnetic resonance imaging to analyze myocardial strain from healthy heart.

Supplementary movie 2. Cine movie of tagging cardiac magnetic resonance imaging to analyze myocardial strain from saline treated heart 22 days after I/R injury.

Supplementary movie 3. Cine movie of tagging cardiac magnetic resonance imaging to analyze myocardial strain from UP-hydrogel treated heart 22 days after I/R injury.
